# Man-Midwifery

**Published:** 1830-10-01

**Authors:** 


### MAN-MIDWIFERY.

1. An important Address to Wives and Mothers on the Dangers and Immorality of Man-Midwifery. By a Medical Practitioner. Octavo, 1830.

2. Eyes for the Blind—Man-midwifery exposed, &c. &c. Octavo, 1830. By M. Adams.

We attach very little importance to the names, or the want of names, in these pamphlets. The harangues of a late President of the College of Surgeons (Sir A. Carlisle) form a sufficient clue to the source whence these detestable and dishonourable pamphlets emanate. The first on the list, from its style, is evidently the production of the collegiate president himself—and as the second (Eyes for the Blind) is addressed to Sir A. Carlisle, Surgeon to the King and Westminster Hospital, there can be little question that it is either the product of the Knight's brains —or that it has been concocted under his immediate auspices.

When a medical man appeals to the public, through the medium of a daily newspaper, on an important medical question, there can be no doubt that his cause is weak—if not bad; but when he descends to the mean quackery, of employing a man to trudge the streets of this metropolis, with a large label on his back, entitled "Eyes for the Blind"—or an "Important Address," &c. we can estimate the state of his intellects, the honesty of his intentions, and the force of his arguments, with a tolerable degree of certainty. We cannot, of course, assert that these pamphlets were written by Sir Anthony Carlisle; but this we can assert, that if the second on the list, dedicated to the Ex-president, be not disowned and repudiated, the dedicatee shares, in no small degree, the disgrace of its "beastly and indelicate" tendency. We shall not occupy our pages, or the time of our readers, with any analysis of these detestable productions, dishonorable alike to the heads and hearts of their authors. Of all the addresses to the worst feelings and prejudices of the ignorant which we have ever read, these pamphlets bear off the palm. The first, for example, commences with "*some parts* of the evidence given, during an inquest held upon the body of a woman who was represented to have died in consequence of improper treatment by a Man-midwife." No reference is given as to the where and when of this coroner's inquest; but because a raw apprentice, in the absence of his master, is accused of unnecessary interference, in dilating the os uteri with his finger—and because the woman died six days after delivery, the inference is charitably drawn, that all mechanical assistance is unnecessary or pernicious —and, consequently, that man-midwifery is a "disgrace to morality and feminine dignity." The above is a specimen of the honesty of the arguments throughout these two productions. We shall record one or two instances in the words of the amiable authors themselves.

"I am acquainted with ladies who have had several children, and who, in some instances, were delivered by females, in others by surgeons of 'considerable eminence.' Now I can easily distinguish those which Messrs. A. and B. *brought* into the world from those Mrs. — received. The former smaller and weakly, displaying distinguishing proofs of the "*men-midwives*" anxiety to welcome the young visitors, while the latter had evidently met with a more tender and friendly, though perhaps less *feeling* reception.

'What is this delicate young gentleman's name? said a noted Surgeon and Phrenologist to a lady of my acquaintance, 'he has the most beautiful craniological head I ever met with—such conscientiousness, such combativeness, but governed by conscientiousness!— and such talent for inquiry,—his organs of causality, locality, and acquisitiveness, are positively the most prominent I ever saw.' 'Why, Mr. ———, have you forgotten Thomas, whom you delivered me of the night you afterwards attended Lady —, shortly before you went to Bath? You surely don't forget how anxious you were, and what pain I suffered, though he is smaller than any of the rest of which Mrs. — has attended me.'

"How much Gall and Spurzheim owe to those—I am at a loss what to term them, 'man-midwives.' They are, in truth, most clever Phrenological bump makers, and if the Cranioscopic system be correct, expecting parents can procure dispositions and characters in variety, by merely informing their *Gentlemen* attendants what temper they wish their offspring to possess; and he will dexterously mould and form the pliant yielding skull to the requisite shape. 'Nurse,' said Mr. Obstetric, 'that child's head is a strange shape, it squints abominably, and seems convulsed.' 'Yes, Sir, and Mistress thinks you hurt it last night, you were so *persevering*.' 'Oh no, my good woman, I shall tell her it is a natural bad formation, and you may wash it often with a little vinegar and warm water, until that *redness* goes off, then you may *press the head gently* into a better form.' This is not an uncommon case, and I have seen instances of obliquity of vision, convulsions, &c. caused most evidently by imprudent fingering. In some of them, the defect and dangers have been removed or moderated by judiciously-applying pressure in such a way as to raise the cause of irritation, the depressed cranium: but this does not always succeed, and unless the naturally good constitution of the child, with careful nursing, overcome the baneful tendency incurred by the attendant's ill-judged method, it either meets a miserable death, or betrays distressing symptoms of bodily deformity or mental weakness."—*Address.*

The foregoing extract from the more moderate of the two pamphlets, and which is generally attributed to the pen of the ex-president himself, will suffice. In the other pamphlet, (EYES FOR THE BLIND) the most palpable falsehoods, the most gross and villainous insinuations, the most detestable and obscene misrepresentations, are scattered in every page. Yet this pamphlet is "*allowed*" to be dedicated to Sir A. Carlisle by the nominal author!! We shall not pollute our pages by more than one short extract from this infamous publication—but "*ex uno disce omnes.*"

"Dr. Power recommends friction upon the parts where the pain is most severe. The doctor is to rub a woman's belly and her back, or forsooth the inside of her thighs, till his arms ache! Nice employment this for a young man full of energy, with lust at his elbow, and a beautiful young woman for his tickling-post!!! Men are but mortal, whether doctors or divines, and such familiarities must require more than human forbearance to exclude criminality."

Such is the bestiality of a pamphlet which Sir A. Carlisle has politely and graciously permitted the author (or pretended author) to dedicate to his knightship! That the sentiments contained in this worthy production are in harmony with those of the EX-PRESIDENT of the College of Surgeons will be rendered more than probable by certain extracts which we shall take the liberty of making from one of Sir Anthony's own manifestos, as published in the TIMES Newspaper, some time ago.

"My Lords and Gentlemen!

"Some months since I addressed a letter, through the medium of this paper, to the King's Secretary for the Home Department, to caution him against the *worldly designs and the injurious practices of men-midwives*, and, if I am rightly informed, those statements have been well received by all the *disinterested and respectable members of the medical profession (!!)* It was to be expected that teachers of man-midwifery, and their adherents, *who regard the healing art chiefly for its profits*, would become outrageous against the persons who expose their *dishonorable vocation*, and accordingly they have been liberal in abuse of them, and loud in the praise of their 'Diana of the Ephesians;' but few sensible men calculated upon a by-intrigue to persuade the public that the birth of mankind ought to be considered a surgical operation; *yet, this absurdity actually disgraces the printed circulars of a College, which possesses the unem-*

*ployed means for taking the highest station in Europe*. It is my firm conviction, that the establishment and the further prevalence of man-midwifery, sanctioned as a branch of surgery, would compromise the justice of the country, *by exposing the lives of child-bed women and infants to many dangerous and unnecessary secret operations*. Under this impression, I should be *passively dishonest*, if I were to neglect the severe duty of asserting my professional thoughts. Having devoted as much time to the study of the *elementary* sciences which constitute the only safe foundation for the healing art as any of my contemporaries, and having, from long-continued meditation and from experience, endeavoured to distinguish the means which help, and those which are hurtful in the perilous business of surgery, I am free to confess that I view *the operations of men-midwives as the most uncertain and the most violent of surgical enterprises*." "That educated men should submit to be associated with *nurses and gossips*, for whole days and nights, merely to wait *the humiliating events of parturition*, is contrary to *decency* and common sense; men-midwives, therefore, teach their disciples to assume directorial offices, and *to be curiously or officiously meddling*, under various pretences, by which the terrified and shocked distressed object is rendered obedient; and when the operator's patience begins to fail, or his predictions are at fault, *he rushes into the perilous adventure of using his conjectural desperate art*; and I confidently believe that the increasing number of deaths to mothers and infants, as well as the pretended difficulties in midwifery, are mainly, if not altogether, *imputable to such undue or improper interference*. Whenever a degree of violence dangerous to the life of a parent or child is meditated, the moral propriety of it should be confided to physicians or hospital surgeons of *enlarged intellect*. My present purpose is, therefore, to awaken the attention of the legal authorities of this kingdom, and to prepare them for *deeds which must arouse the indignation of parties who may suffer from the audacity of young adventurers in surgical midwifery*. Even before this innovation, it cannot be denied that many rash surgeons have been hurried by vanity, or from pecuniary necessity, urged to seek premature vulgar fame, by attempting unjustifiable operations, trusting that fatal results would be hushed for the sake of the character of the profession, and my own experience in a metropolitan general hospital, where every medical officer is kept in check by rivals, has induced me to hold public consultations in the presence of all the students, in order to prevent *questionable enterprises*. If such precaution is needful in public practice, what security can we find in the privacy of a lying-in-room, where often none but ignorant women are present, and where surgical acts of violence may be passed over without inquiry? The public are not aware that the self-constituted teachers of what is now termed 'The Obstetric Art and Science,' are *not any of them general hospital surgeons, or hospital physicians*, and their assumed authority to dictate to surgeon's pupils the terms on which they may *commit irremediable injuries to women, or destruction to infants*, are not sanctioned by law. I do not announce these alarming statements unadvisedly, but from serious apprehensions, awakened by the flippancy with which men-midwives write and speak of sacrificing a child, or wounding the *vital parts* of a mother." "I, therefore, most respectfully submit, that whenever cases of violent death occur to mother or infant, from the use of surgical instruments or surgical hands, a coroner's inquest should be holden, and if sufficient proofs are adduced of hasty violence, or of rashness, the affair should be investigated before a jury, and a chief reliance placed upon the opinions of *some grave disinterested physician, or experienced hospital surgeon*, they being persons the best qualified to understand the intricate hinges of life or death, and to determine how far it may be ever expedient, under given circumstances, to hazard the life of a mother, or that of her progeny."—A. Carlisle.

That a medical man, who has held the distinguished and honorable posts of President of the College of Surgeons, and surgeon to a metropolitan hospital, should give utterance to such sentiments as are contained in the foregoing extracts—and that in a public newspaper, we could not have believed, had we not read them twice over, and found that they were not disavowed as a forgery.

When we contemplate the opprobrious epithets and the criminal accusations which this man lavishes on his brethren, and when we reflect that there is not one atom of fact for their foundation, we are at some loss to account for the motives which could have led him to the publication of such slanderous insinuations. We do not think it would be unfair to attribute such malicious inventions to depraved thoughts passing in his own mind. But we will not be so uncharitable as the writer of the foregoing epistle. We attribute the sentiments therein contained to two sources—an obliquity of intellect—and a temper soured by disappointment. That any man whose senses were not warped, and whose judgment was incapable, on this subject, of drawing rational conclusions from obvious facts, could have indited such an epistle, or dared to insinuate such criminal conduct against three-fourths—nay, nine-tenths of the medical profession, we fearlessly deny:—while the tone in which his animadversions are conveyed, indicate the other part of our proposition, the soured temper. If the author has devoted so much time to "elementary sciences," to "long-continued meditation," to "experience," &c. he has turned his time and talents to little account, when he is led to "view the operations of men-midwives as the most *uncertain* and the most *violent* of surgical enterprizes." Where did Sir Anthony learn these things? He never practised midwifery himself, and yet he has the presumption to decide on the dangers, the violence, and the injuries occasioned by the obstetric art!! This utter ignorance leads him to pronounce that decency is outraged by the attendance of surgeons on parturient women; whereas, all who know any thing of the matter, are aware that the attendance of females is the source—the fertile source of indecency. Sir Anthony's insinuation respecting "unnecessary *secret* operations"—"wounding the *vital parts* of a mother," &c. are so abominable and diabolical, that we would not be author of them for all the gems of Golconda's mines. If the writer of the foregoing letter occupied the highest pinnacle of fame in the medical profession, it would level him in the dust;—as it is, the author has not much to fear from the depth of the fall.

